# Impact of Chromosomal Rearrangements on the Interpretation of Lupin Karyotype Evolution

**DOI:** 10.3390/genes10040259

**Published:** 2019-04-01

**Authors:** Karolina Susek, Wojciech Bielski, Katarzyna B. Czyż, Robert Hasterok, Scott A. Jackson, Bogdan Wolko, Barbara Naganowska

**Affiliations:** 1 Department of Genomics, Institute of Plant Genetics, Polish Academy of Sciences, 60-479 Poznan, Poland; wbie@igr.poznan.pl (W.B.); bwol@igr.poznan.pl (B.W.); bnag@igr.poznan.pl (B.N.); 2 Department of Biometry and Bioinformatics, Institute of Plant Genetics, Polish Academy of Sciences, 60-479 Poznan, Poland; kwyr@igr.poznan.pl; 3 Department of Plant Anatomy and Cytology, University of Silesia in Katowice, 40-032 Katowice, Poland; robert.hasterok@us.edu.pl; 4 Center for Applied Genetic Technologies, University of Georgia, Athens, GA 30602, USA; sjackson@uga.edu

**Keywords:** *Lupinus*, lupin, chromosome, pseudomolecule, cytogenomic map, karyotype structure, evolution, BAC, FISH, comparative chromosome mapping

## Abstract

Plant genome evolution can be very complex and challenging to describe, even within a genus. Mechanisms that underlie genome variation are complex and can include whole-genome duplications, gene duplication and/or loss, and, importantly, multiple chromosomal rearrangements. Lupins (*Lupinus*) diverged from other legumes approximately 60 mya. In contrast to New World lupins, Old World lupins show high variability not only for chromosome numbers (2n = 32–52), but also for the basic chromosome number (x = 5–9, 13) and genome size. The evolutionary basis that underlies the karyotype evolution in lupins remains unknown, as it has so far been impossible to identify individual chromosomes. To shed light on chromosome changes and evolution, we used comparative chromosome mapping among 11 Old World lupins, with *Lupinus angustifolius* as the reference species. We applied set of *L. angustifolius*-derived bacterial artificial chromosome clones for fluorescence in situ hybridization. We demonstrate that chromosome variations in the species analyzed might have arisen from multiple changes in chromosome structure and number. We hypothesize about lupin karyotype evolution through polyploidy and subsequent aneuploidy. Additionally, we have established a cytogenomic map of *L. angustifolius* along with chromosome markers that can be used for related species to further improve comparative studies of crops and wild lupins.

## 1. Introduction

Knowing how genomes are organized and distinct at the chromosomal level is fundamental for an understanding of the dynamics of chromosomal structure and karyotypes [1]. Chromosome number variations have been studied as an intermediary of karyotype changes. Traditionally, chromosome numbers, genome size, and the number of nucleolar organizers have been used to establish the basis for further studies of chromosome organization, especially when data on whole genome sequences or differential chromosome markers are not available. Progress in molecular cytogenetics and the availability of bacterial artificial chromosome (BAC) libraries have advanced plant genome studies [2], to provide more precise identification of karyotypes and to subsequently integrate these with linkage groups [3,4]. Whole-genome sequencing was a breakthrough that has improved the quality and accuracy of genome maps, and has made it possible to assign chromosomes to pseudomolecules in various plant species [5,6,7]. Importantly, reference karyotypes have become a key tool for comparative chromosome mapping, e.g., among the legume genera *Arachis* [8], *Glycine* [9], and *Phaseolus* [10,11], as well as the genera of other families, including *Solanum* [12] and *Daucus* [13]. 

Plants of the *Lupinus* genus (lupins; Genisteae tribe) belong to the important grain legumes, along with those of the genera *Glycine*, *Phaseolus*, and *Arachis*. Lupins are a source of valuable protein, oils, and bioactive compounds, such as alkaloids, isoflavones, and oligosaccharides. Additionally, lupins contribute to the productivity and quality of agricultural soils, and thus they have substantial roles in crop rotation. The *Lupinus* genus consists of approximately 270 species, which can be divided into two groups, known as Old World lupins (OWLs) and New World lupins (NWLs). OWLs comprise 12 autogamous species, which include three crops: *Lupinus angustifolius* (narrow-leafed lupin), *Lupinus albus* (white lupin), and *Lupinus luteus* (yellow lupin) [14]. The OWLs are geographically and phenotypically diverse, and they are mainly represented in Mediterranean regions and North Africa, which highlights their adaptation to different environments. Based on their seed coat structure, the OWLs are traditionally divided into two groups. The first group includes the smooth-seeded lupins, which is divided into four sections: Angustifolius (2n = 40), Luteus (2n = 52), Albus (2n = 50), and Micranthus (2n = 52). These are distributed around the Mediterranean basin, and there are major genetic barriers between most species. Phylogenetic systematics using internal transcribed spacer data have shown that smooth-seeded species include at least two lineages, i.e., *Angustifolius–Luteus* and *Micranthus–Albus* [15]. The second group of the OWLs comprises the rough-seeded lupins, which have been divided into three sections: Pilosus (2n = 42), Atlanticus (2n = 32, 36, 38) and Princei (2n = 38). These are eco-geographically isolated and are mainly in North Africa. 

At the chromosome level, OWLs are a complex group within legumes. They have chromosome numbers that range from 2n = 32 to 2n = 52, and a varied basic chromosome number x = 5 − 9, 13 [16], which indicates an obscure evolutionary pattern, as has been exemplified across *L. angustifolius* and *Lupinus cryptanthus*, *Lupinus micranthus*, *Lupinus pilosus*, and *Lupinus cosentinii* [17]. Recently, differences in chromatin modification and DNA methylation have also highlighted variations in the organization of the lupin genomes [18]. Progress in genetic and molecular studies in lupins has mostly arisen from crops, and has been linked, for example, with the development of genetic maps [19], whole-genome sequencing [20,21], and transcriptomic analyses [22]. Of note, *L. angustifolius* and its genomic resources is considered as the reference species in comparative studies. The assignment of chromosomes to linkage groups of *L. angustifolius* [23,24] established the starting point for the tracking of cytogenetic differences among crops and wild lupins. 

Here, we present the first complete genome-based cytogenetic map of *L. angustifolius*. For this purpose, we assigned the reference karyotype [24] to its whole genome assembly [20]. We explored the possibilities of its application in lupin genome studies by providing the first chromosome markers for related species. Finally, we report on a first global view of the chromosome structure in lupin karyotypes and assume their complex patterns of evolutionary changes. A considerable part of this study was addressed to the fundamental question of whether a common model of lupin karyotype evolution exists, according to the most likely explanations of the observations. Our data provide a foundation for lupin chromosome and karyotype evolution studies.

## 2. Materials and Methods

### 2.1. Plant Material

Twelve species of both crop and wild lupins were used in this study: The crop *L. angustifolius* and its wild botanical form *L. cryptanthus*; the crop *L. luteus* and *Lupinus hispanicus*; the crop *L. albus* and its wild form *Lupinus graecus*; and six other wild lupins. Their names, basic taxonomy, and cytogenetic characteristics are provided in Table 1. Their origins and geographic distributions are shown in Supplementary Figure S1.

The seeds were germinated in Petri dishes at 25 °C, to obtain root tips that were suitable for mitotic chromosome preparations.

### 2.2. BAC DNA Isolation, Sequencing, and Labeling

BAC clones were originated from the genomic BAC library constructed by Kasprzak et al. [25]. The BACs used for assignment of linkage groups to chromosome of *L. angustifolius* [23,24] and other available in NCBI database are listed in Table 2 (Results section). BAC DNA was isolated using standard miniprep kits (QIAprep Spin; Qiagen, Hilden, Germany). BAC DNA sequencing was carried out using the long-read sequencing (PacBio), as described by Susek et al. [17], or by the next-generation sequencing (NGS) approach (Illumina) [24]. Whole BAC sequences (WBS) were imported into Geneious 8.1.6. (Biomatters, Ltd., Auckland, New Zealand) and aligned to the *L. angustifolius* pseudomolecules and/or scaffolds of Hane et al. [20]. The BAC-end sequences (BES) were also used when the WBS were not available. For generation of the fluorescence in situ hybridization (FISH) probe, BAC DNA was labeled by nick translation (Sigma-Aldrich, St. Louis, MI, USA), either with digoxygenin-11-dUTP (Sigma-Aldrich), or with tetramethylrhodamine-5-dUTP (Sigma-Aldrich).

### 2.3. Fluorescence In Situ Hybridization

Mitotic chromosome preparations and FISH using the BAC-based probes (BAC-FISH) were carried out according to Lesniewska et al. [23] for *L. angustifolius*, and as described by Susek et al. [17] for other lupins, with minor modifications. Root tips were treated in an enzyme solution of 40% (*v*/*v*) pectinase (Sigma-Aldrich), 3% (*w*/*v*) cellulase (Sigma-Aldrich), and 1.5% (*w*/*v*) cellulase ‘Onozuka R-10’ (Serva, Heidelberg, Germany), at 37 °C for 150 min for the primary roots of the crops, and for 60 min to 90 min for both the primary and the lateral roots of the wild lupins. The same set of BAC markers was used for all of the species in a given experiment, to maintain the same conditions of the BAC-FISH reaction. To localize the BAC-FISH signals, the chromosomes were counterstained with 4′,6-diamidino-2-phenylindole (DAPI) in Vectashield (Vector Laboratories, Burlingame, CA, USA).

### 2.4. Microscopy, Image Acquisition and Processing, Data Presentation

The FISH data were analyzed using an epifluorescence microscope (BX-60; Olympus, Tokyo, Japan). Images were acquired with a high-sensitivity monochromatic camera (Ikegami 47E), separately for each of three fluorochromes, using the appropriate excitation and emission filters. Grey images were pseudocolored (Wasabi; Hamamatsu Photonics v. 1.1.) and superimposed (Micrografx; Corel Picture Publisher 10.1 software). At least five images were analyzed to confirm the intensities and chromosomal distributions of the individual BAC-FISH signals. All of the lupin karyotypes were visualised (ChromDraw package; included in Bioconductor R packages; www.bioconductor.org/packages), as described by Janečka and Lysak [26].

## 3. Results

### 3.1. Assignment of Chromosomes to Pseudomolecules of the L. angustifolius Genome

Physical mapping of chromosomes is a robust and efficient way for comparative studies of karyotype structure and evolution based on the synteny and collinearity of related genomes. As a framework, we used the cytogenetic map of *L. angustifolius* that was developed by Wyrwa et al. [24] to assign the *L. angustifolius* chromosomes to pseudomolecules [20]. Different abbreviations are used for chromosome names in the literature, such as LANG [23] and Lang [24], while both linkage groups and pseudomolecules are named as NLL. Here, for clarity, we use the following abbreviations: Lang for *L. angustifolius* chromosomes, and NLL for pseudomolecules. As a consequence of this assignment, we propose to name the chromosomes and pseudomolecules as Lang01 to Lang20. 

We selected 53 BAC clone-based cytogenetic markers of *L. angustifolius* that were previously generated by Lesniewska et al. [23] and Wyrwa et al. [24] to assign the chromosomes to linkage groups of *L. angustifolius* (see detailed data in Supplementary Table S1). Among these, there were some BAC clones (i.e., 1M23, 2B03, 8C03) that remained unassigned. We sequenced 22 BACs, as 18 by long-read sequencing (PacBio) and four with an NGS platform (Illumina). These resources were used to accurately integrate the chromosomes and pseudomolecules in the Lang vs. NLL map of *L. angustifolius*. This set of new WBS, along with other lupin BAC sequences available at the NCBI database, established efficient tools for reciprocal integration of chromosomes and pseudomolecules (Table 2). We assigned Lang vs. NLL using varied numbers of WBS for particular chromosomes: Five WBS for Lang06; four for Lang 08 and 13; three for Lang 03, 11, 17, and 20; and two for Lang 05, 10, 14, and 19. For Lang 01, 02, 04, 07, 09, 12, 15, 16, and 18, we used one WBS. In the cases where WBS and/or BES provided misassembled information between chromosomes and pseudomolecules, we used BAC-FISH to integrate the *L. angustifolius* chromosomes and pseudomolecules. As a consequence, seven chromosomes were re-assigned (Table 2, Lang 01, 07, 08, 13, 14, 19, 20).

**Figure 1:**
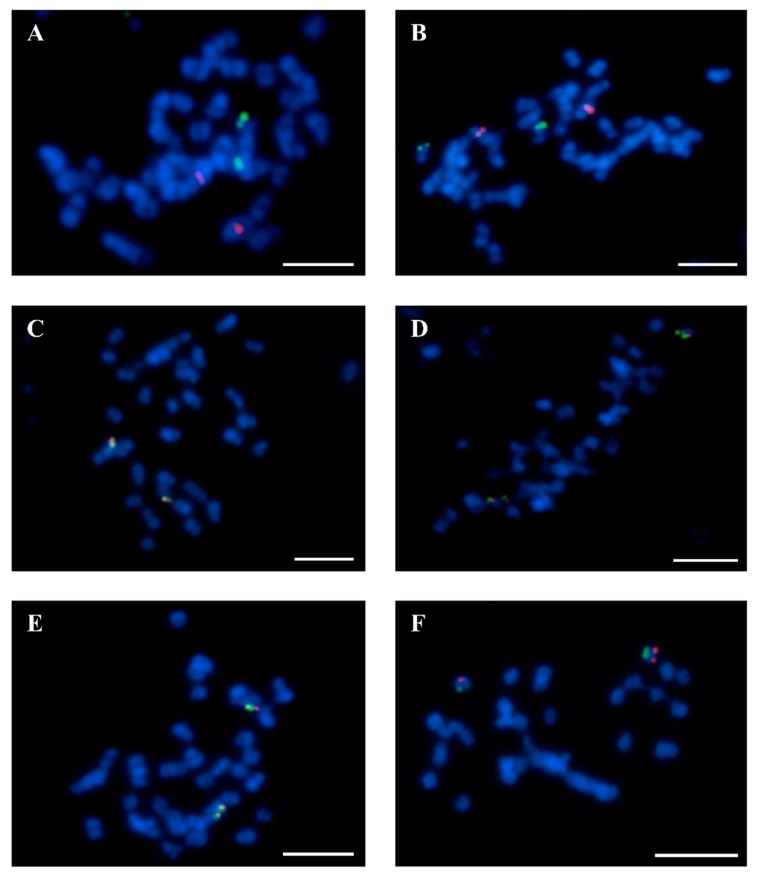
Fluorescence in situ hybridization verification of BAC localization in chromosomes of *L. angustifolius*. (**A**) BAC 43C18 (red signals)—Lang 01 and BAC 51F15 (green signals)—Lang 13. (**B**) BAC 2B03 (red)—Lang07 and 74I10 (green)—Lang19. (**C**) 67C07 (red)—Lang19 and 74I10 (green)—Lang19. (**D**) BAC 11G20 (red)—Lang13 and BAC 51F15 (green)—Lang13. (**E**) BAC 51F15 (red)—Lang13 and 8C03 (green)—Lang13. (**F**) 17B07 (red)—Lang20 and 1M23 (green)—Lang20. Chromosomes were stained with DAPI (blue). Scale bars = 5 µm.

**Figure 2:**
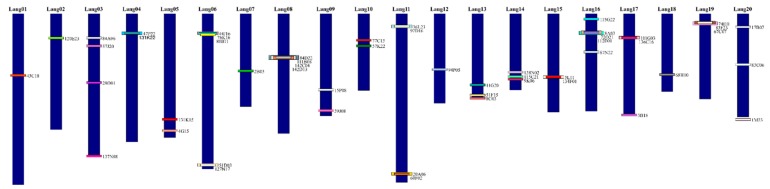
Ideogram of *L. angustifolius* chromosomes (Lang01 to Lang20). The karyotype was created based on reciprocal assignment to the whole genome of *L. angustifolius*. The BAC clones are indicated by rectangles with a unique color, according to their position in the genome. The overlapping rectangles are shown overlapping BAC positions in *L. angustifolius*. All of the chromosomes are drawn to scale, whereby the Mb units refer to the *L. angustifolius* genome sequence.

The chromosome Lang01 was verified by BAC 43C18, although WBS 43C18 was identified in both pseudomolecules NLL01 and NLL13. BAC-FISH mapping precluded assignment to NLL13 (Figure 1A, Table 2). Interestingly, Lang07 was previously marked by the clone 74I10, and has now been anchored by BAC 2B03 on a different chromosome to BAC 74I10 (Figure 1B). Additionally, the alignment of 74I10 WBS to the whole genome sequence, along with BAC-FISH mapping, defined this clone as a marker for Lang19 (Figure 1C). Lang19 was described by two other BACs, 67C07 and 83F23, with the WBS of 67C07 positioned in NLL19 (Table 2). Unexpectedly, four BACs (84D22, 111B08, 142C04, 142D13) were assigned to Lang08, even though they all corresponded to NLL17 (Table 2). Furthermore, we verified that BACs 11G20, 8C03, and 51F15 belong to the same chromosome Lang13, although 11G20 WBS was aligned to many NLLs (Table 2). BAC-FISH using both clones 11G20 and 51F15 (Figure 1D) showed that these BACs belong to the same chromosome Lang13. Moreover, Lang13 was enriched with the marker 8C03, assigned to chromosome Lang13 (Figure 1E). Similar to Lang13, Lang14 with clones 138N02, 115C21, and 9K06 illustrated some miss-mapping, such as the alignment of 5′BES and 3′BES of 115C21 to two different pseudomolecules, Lang14 and Lang12, respectively (Table 2). Nevertheless, BAC-FISH with 9K06 and 115C21 confirmed that Lang14 corresponded to NLL14 (not shown). Within Lang16, five BACs were identified (115G22, 112E01, 72O21, 8A03, 87N22) in various NLLs based on BESs (Table 2). However, the whole sequence of BAC 87N22 was in NLL16. Considering that all of these BACs were mapped to the same chromosome by Wyrwa et al. [24], all five BACs were linked to the same chromosome, Lang16. Our integrated studies also led to the assignment of clone 1M23 from cluster-2 to pseudomolecule NLL20 (Table 2), and to its colocalization with BAC 17B07, which is a marker of chromosome Lang20 (Figure 1F). Finally, 13 chromosomes (i.e., Lang02–06, Lang09–12, Lang15, Lang16–18) were assigned to pseudomolecules of *L. angustifolius*, in agreement with former chromosome assignments to linkage groups [23,24]. Finally, combined analyses of multiple BAC-FISH reactions, BAC sequencing, and whole genome assignment led us to construct the first comprehensive ideogram of the *L. angustifolius* chromosomes (Figure 2). 

### 3.2. Chromosome Variation in Lupins

**Figure 3:**
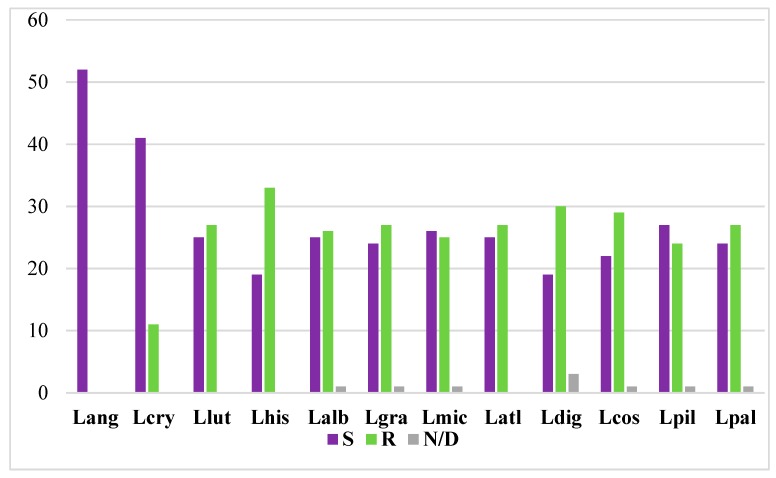
Representation of ‘single’ (S) and ‘repetitive’ (R) BAC clones in 11 lupin genomes. Lang, *L. angustifolius* (reference species). Lcry, *L. cryptanthus*. Llut, *L. luteus*. Lhis, *L. hispanicus*. Lalb, *L. albus*. Lgra, *L. graecus*. Lmic, *L. micranthus*. Latl, *L. atlanticus*. Ldig, *L. digitatus*. Lcos, *L. cosentini*. Lpil, *L. pilosus*. Lpal, *L. palaestinus*. N/D, not detected.

The ideogram obtained for the *L. angustifolius* chromosomes (Figure 2) provides the foundation for comparative cytogenomic mapping among lupins. Such chromosome analyses have been conducted using heterologous BAC-FISH mapping among 11 species of the genus: *L. cryptanthus* (2n = 40, Lcry), *L. luteus* (2n = 52, Llut), *L. hispanicus* (2n = 52, Lhis), *L. albus* (2n = 50, Lalb), *L. graecus* (2n = 50, Lgra), *L. micranthus* (2n = 52, Lmic), *L. atlanticus* (2n = 38, Latl), *Lupinus digitatus* (2n = 36, Ldig), *L. cosentinii* (2n = 32, Lcos), *L. pilosus* (2n = 42, Lpil), and *Lupinus palaestinus* (2n = 42, Lpal). We comparatively mapped 52 BAC-clone-based chromosome markers of *L. angustifolius* karyotype to provide cytogenetic landmarks for related lupins. These BACs were either mapped to one unique locus (so-called ‘single’ BAC; indicated as S here) or to many loci along the chromosomes of the analyzed lupins (referred to as ‘repetitive’ BAC; indicated as R here). Particular clones were mapped either as S or R markers; e.g., clone 84A06 was mapped to one locus in *L. cryptanthus*, *L. albus*, *L. graecus*, *L. micranthus*, *L. cosentinii*, *L. pilosus*, and *L. palaestinus*, but had many loci in the remaining lupins. Their FISH patterns are summarized in Table 3. Among the 52 clones, we have provided a set of 41 markers for Lcry, 25 for Llut, 19 for Lhis, 25 for Lalb, 24 for Lgra, 26 for Lmic, 25 for Latl, 19 for Ldig, 22 for Lcos, 27 for Lpil, and 24 for Lpal (Figure 3). Eight BACs (i.e., 80B11, 59J08, 115G22, 112E01, 72O21, 8A03, 136C16, 17B07) were shown to be unique markers for all lupins. 

The set of markers for each species led us to identify chromosomes in the 11 OWLs analyzed (Supplementary Figures S2–S12). These had varied numbers of Lang-like chromosomes—19 in Lpil (Supplementary Figure S11); 18 in Lmic (Supplementary Figure S7); 17 in Lcry (Supplementary Figure S2); 16 in Llut (Supplementary Figure S3) and Lcos (Supplementary Figure S10); 14 in Lalb (Supplementary Figure S5), Lgra (Supplementary Figure S6), and Lpal (Supplementary Figure S12); 13 in Latl (Supplementary Figure S8); 12 in Ldig (Supplementary Figure S9); and 11 in Lhis (Supplementary Figure S4). Due to no reference information about other lupin genomes, the chromosome sizes given here were based on *L. angustifolius*. Also, the chromosomes of the lupins studied were numbered according to the chromosomes of the reference species; e.g., Lcry02 corresponds to Lang02, and so on.

The ‘repetitive’ BACs were excluded from the chromosomal marker analyses, although they broaden our view about chromosome variations in lupins. Combining the S and R clones, we illustrated Lang-like chromosomes in related species, to identify different levels of synteny between the lupins (Supplementary Figure S13). We identified 11 R clones for Lcry, 27 for Llut, 33 for Lhis, 26 for Lalb, 27 for Lgra, 25 for Lmic, 27 for Latl, 30 for Ldig, 29 for Lcos, 24 for Lpil, and 27 for Lpal (Figure 3). Three clones were not detected in Ldig, and another one was not identified in Lalb, Lgra, Lmic, Lcos, Lpil, and Lpal (Table 3). Additionally, we estimated that eight clones (43C18, 28O01, 15P08, 11G20, 115C21, 5L11, 87N22, 68H10) hybridized to many loci in the chromosomes of all of the lupins analyzed. On the other hand, all of the clones from Lang 05, 11, and 13 were ‘repetitive’ only for Lhis, Ldig, and Lpal, respectively.

**Figure 4:**
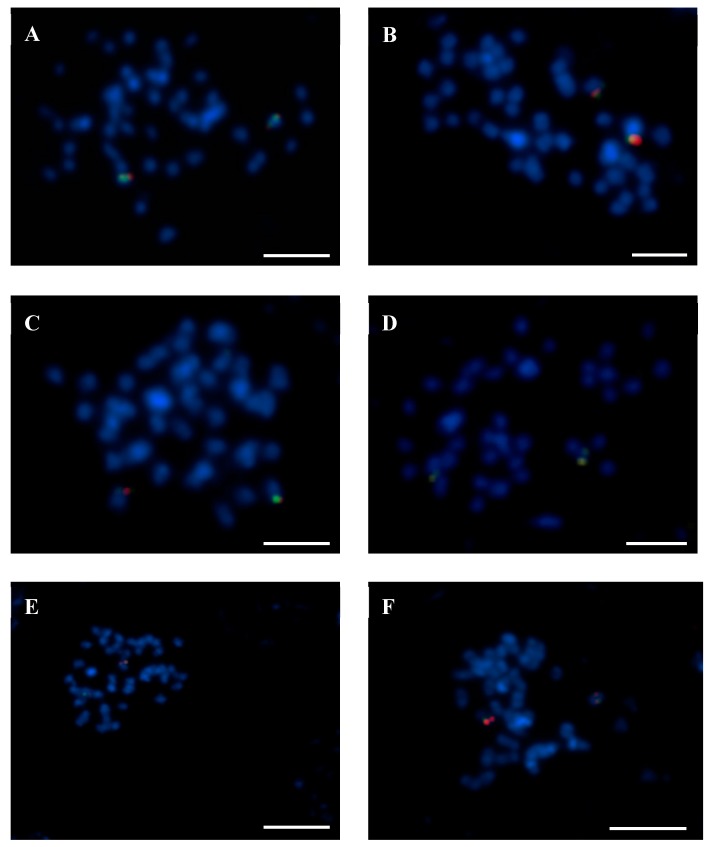
Identification of the Lang05-like chromosome in related lupins using fluorescence in situ hybridization (FISH) with BAC 131K22 (red signals) and BAC 4G15 (green signals). (**A**) *L. albus*. (**B**) *L. pilosus*. (**C**) *L. palaestinus*. (**D**) *L. atlanticus*. (**E**) *L. digitatus*. (**F**) *L. cosentinii*. Chromosomes were stained with DAPI (blue). Scale bars = 5 µm.

Based on the FISH patterns for both ‘single’ and ‘repetitive’ BACs, we distinguished four types of Lang-like chromosomes in related species. The first reflects the most varied BAC-FISH pattern, as Lang 03, 04, 06, 08, 10, 11, 13, 17, 19, and 20 illustrated that BACs from particular chromosomes were localized differently in most species. The second type was recognized as the most conserved pattern, e.g., two clones of Lang05 were localized as S in the chromosomes across all species, with the exception of *L. hispanicus* (for an example of comparative BAC-FISH mapping, see Figure 4). Moreover, BACs 15P08 and 59J08 from Lang09 were mapped as R and S in all of the lupins. Also, clones of Lang16 were mapped with the same pattern in all of the lupins. The third type comprised five Lang-like chromosomes, e.g., Lang02, where BAC 120E23 was mapped as S in most lupin species, except for *L. albus*, *L. graecus*, and *L. digitatus*, and Lang07, where the clone 2B03 was mapped as R only in *L. micranthus* and *L. digitatus*. Additionally, Lang14 and Lang15 showed two kinds of BAC-FISH patterns. The Lang12-like chromosomes were also included with this type, as the clone 94P05 was mapped as R in seven species, with the exceptions of *L. luteus*, *L. albus*, *L. graecus*, and *L. digitatus*. In Lang14, we recognized three clones as R in five species. In the other five lupins, two clones (138N02, 115C21) were mapped as R, while BAC 9K06 was mapped as S. Exceptionally, for *L. cryptanthus*, 138N02 and 9K06 were mapped as S, but BAC 115C21 hybridized as R. The same pattern was also noted for Lang15, where both clones of this chromosome were R in six lupins, but clone 5L11 and BAC 134F01 were seen as R and S, respectively, in five species. The last type of chromosomes contains the clones represented as ‘repetitive’ in Lang01-like and Lang18-like chromosomes in all species.

Surprisingly, Lang17 was covered by BAC markers that all were ‘single’ in the lupins analyzed, with two exceptions as *L. cryptanthus* and *L. palaestinus*, where BACs 111G03 and 3B118, respectively, were mapped as R. We also found that ‘repetitive’ clones enabled us to distinguish Lang10-like chromosome between crop lupin and its wild form, e.g., marker 77C13 mapped as S in Lalb and R in Lgra. This was similar to the Lang19-like chromosomes of Llut and Lhis, where clone 83F23 was mapped as R and S, respectively. Interestingly, two other clones from Lang19-like chromosomes were mapped with the same pattern (Table 3). 

### 3.3. Chromosome Rearrangements in Lupins

**Figure 5:**
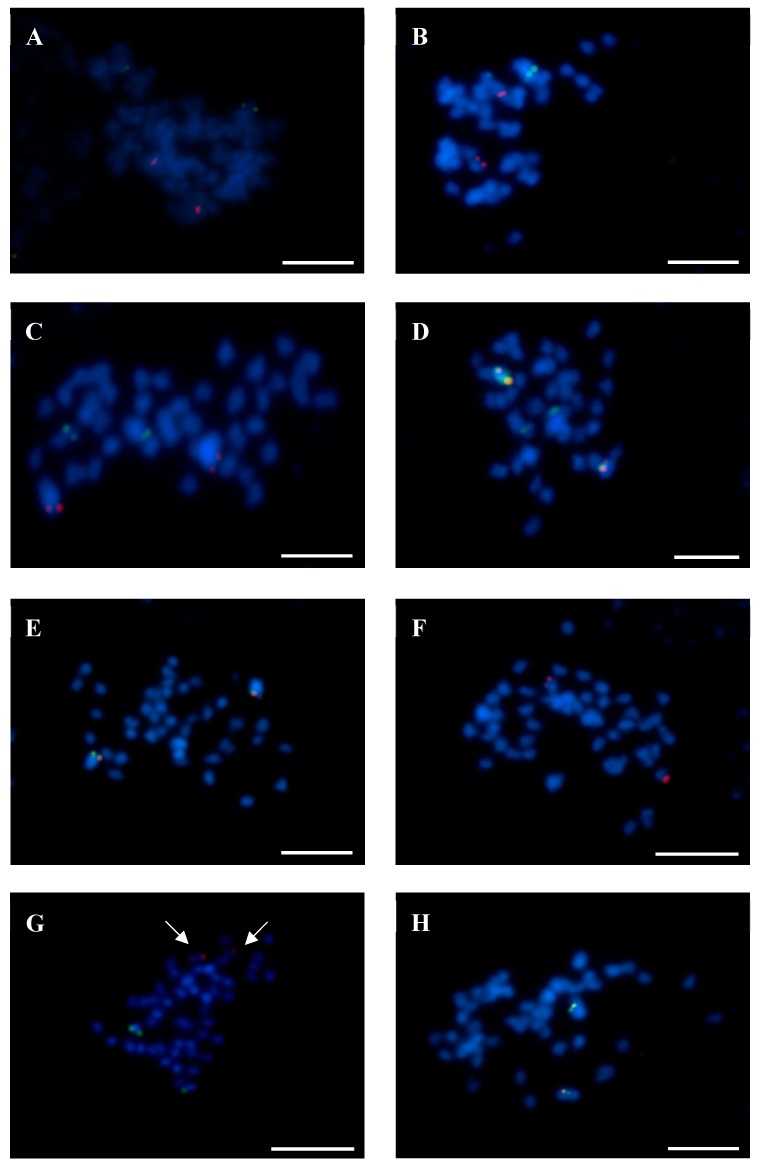
Localization of BAC clones from chromosome Lang06 in chromosomes of related lupins. (**A**) *L. luteus*—BAC 44J16 (red signals) and BAC 51D03 (green signals). (**B**) *L. pilosus*—BAC 80B11 (red) and BAC 76K16 (green). (**C**) *L. pilosus*—BAC 51D03 (red) and BAC 76K16 (green). (**D**) *L. palaestinus*—BAC 80B11 (red) and BAC 76K16 (green). (**E**) *L. digitatus* – BAC 80B11 (red) and BAC 76K16 (green). (**F**) *L. hispanicus*—BAC 80B11 (red). (**G**) *L. micranthus* – BAC 80B11 (red) and BAC 127N17 (green). (**H**) *L. micranthus*—BAC 51D03 (red) and BAC 127N17 (green). Chromosomes were stained with DAPI (blue). Arrows in (H) show the localization of BAC 80B11 (red). Scale bars, 5 µm.

**Figure 6:**
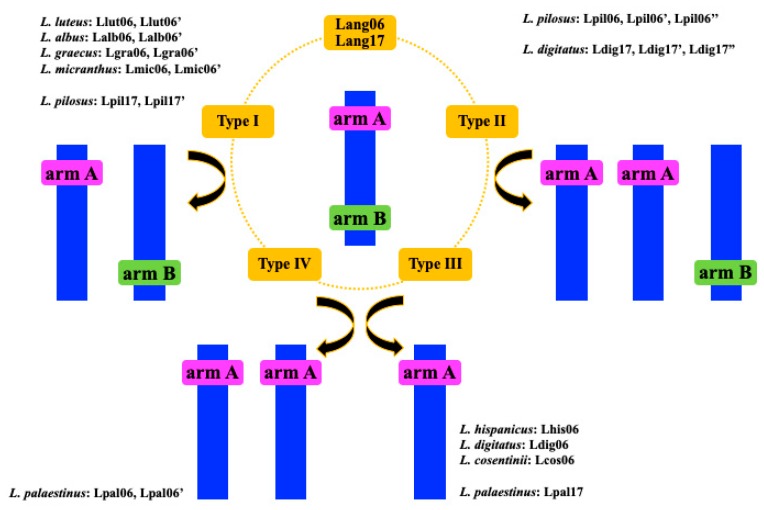
Scheme of the structural rearrangements within Lang06 and Lang17 of *L. angustifolius* and the related lupins. The pink and green rectangles correspond to arm A and arm B of both of the Lang06 and Lang17 chromosomes in related lupins.

Based on comparative analysis of chromosome variations, we distinguished two main types of chromosome rearrangements. The first type consists of structural changes that lead to either increases or decreases in chromosome numbers and involve chromosomes Lang06-like and Lang17-like. We tracked Lang06 based on five clones (44J16, 76K16, 80B11, 51D03, 127N17) that marked the two chromosome arms (here named as arm A, carrying clones 44J16, 76K16, and 80B11, and arm B, with BACs 51D03 and 127N17). We observed different mapping patterns (Figure 5), e.g., clones 44J16 and 80B11 hybridized to one chromosome of *L. luteus* (Llut06), and BACs 76K16 and 80B11 to one chromosome of *L. albus* (Lalb06) and *L. graecus* (Lgra06). However, clone 51D03 was identified on another chromosome (additional chromosomes were marked by ′ or ′′) of these three species, referred to as Llut06′ (e.g., Figure 5A, Supplementary Figure S3, karyotype of Llut), Lalb06′ (Supplementary Figure S5, karyotype of Llalb), and Lgra06′ (Supplementary Figure S6, karyotype of Lgra). In *L. pilosus*, clones 76K16 and 80B11 were mapped in two different chromosomes (Figure 5B), as Lpil06 and Lpil06′, respectively. In addition, clone 51D03 together with BAC 127N17 were mapped in different chromosomes to Lpil06 (for an example of multiple BAC-FISH reactions, see Figure 5C) and Lpil06’, named as Lpil06′′ (Supplementary Figure S11, karyotype of Lpil). In *L. palaestinus*, we observed two chromosomes that carried clones only from one arm of Lang06, one called Lpal06 that hybridized with both BACs 76K16 and 80B11, and the other called Lpal06′ that hybridized with the clone 76K16 (Figure 5D, Supplementary Figure S12, karyotype of Lpal). Of note, one chromosome arm of Lang06 was also detected in Lhis (Supplementary Figure S4, karyotype of Lhis), Ldig (Supplementary Figure S9, karyotype of Ldig), and Lcos (Supplementary Figure S10, karyotype of Lcos), with the difference that Ldig06 carried both clones 76K16 and 80B11 (Figure 5E), while Lhis06 (e.g., Figure 5F) and Lcos06 carried only BAC 80B11. On the other hand, in *L. micranthus*, we visualized two chromosomes (Figure 6G,H) that corresponded to one chromosome Lang06. In this species, clones 76K16 and 80B11 and clones 51D03 and 127N17 were mapped in two chromosomes, Lmic06 and Lmic06′, respectively (Supplementary Figure S7, karyotype of Lmic). The entire set of markers of Lang06 was identified in the Lcry06 chromosome of *L. cryptanthus* and the Latl06 chromosome of *L. atlanticus*. However, in *L. atlanticus*, only BAC 127N17 was mapped, which is one of two clones that originated from the same arm of Lang06 (Supplementary Figure S2, karyotype of Lcry; Supplementary Figure S8, karyotype of Latl). 

We also found that clones 111G03, 136C16, and 3B18 of Lang17 mapped differently in the lupins analysed. In *L. digitatus*, three chromosomes carried these BACs, of which chromosome Ldig17 carried only BAC 3B18. However, clone 136C16 was mapped in another two chromosomes, in Ldig17’ and Lang17”, together with clone 111G03. In *L. pilosus*, two chromosomes were identified by clones that originated from Lang17. Clone 3B18 was the only one that mapped in chromosome Lpil17, while both 111G03 and 136C16 hybridized to chromosome Lpil17’. In *L. palaestinus*, one chromosome Lpal17 was marked by clones 111G03 and 136C16. Finally, in another seven lupin species (*L. luteus*, *L. hispanicus*, *L. albus*, *L. graecus*, *L. micranthus*, *L. atlanticus*, *L. cosentinii*), the BAC-FISH pattern of Lang17-like chromosome revealed its similarity to Lang17, with the exception of *L. cryptanthus*, where Lcry17 carried only two clones (3B18, 136C16).

The second group of chromosomal rearrangements involved insertions and/or deletions, as reflected by the differences in the BAC presence/absence in a given chromosome (for example, see Supplementary Figure S14). We found that eight chromosomes (Lang03–04, Lang08, Lang10–11, Lang13, Lang19–20) showed the greatest changes in the BAC-FISH patterns. All of these three clones (84A06, 57J20, 137N08) from Lang03 were mapped in *L. cryptanthus*, *L. albus*, and *L. palaestinus*, in agreement with *L. angustifolius* (Supplementary Figure S13). However, clones 84A06 and 57J20 were localized in the same chromosome in *L. graceus* (Lgra03), with 84A06 and 137N08 in *L. micranthus* (Lmic03), and *L. cosentinii* (Lcos03) and *L. pilosus* (Lpil03) (Supplementary Figure S13). In the case of Lang08, we determined that clone 111B08 was mapped together with either BAC 142D13 in *L. luteus* (Llut08) or 84D22 in *L. cosentinii* (Lcos08) and *L. pilosus* (Lpil08). The clones 84D22 and 142C04 were colocalized in one chromosome in *L. micranthus* (Lmic08) and *L. cosentinii* (Lcos08) (Supplementary Figures S7, S10). Additionally, we found another chromosome (Lmic08’) that carried BAC 111B08 in *L. micranthus* (Supplementary Figure S7). Tracking the localization of clones from Lang11 in related OWL species, we noted that one (BAC 36L23) of four clones from chromosome Lang11 was detected in Lang11-like chromosomes of *L. luteus*, *L. micranthus*, *L. cosentinii*, and *L. pilosus* (Supplementary Figures S3, S7, S10, S11). The second, BAC 97D16, was mapped in *L. atlanticus* (Latl11) and *L. palaestinus* (Lpal11) (Supplementary Figures S3, S7, S10, and S11). Additionally, clone 97D16 was identified along with clone 36L23 in *L. hispanicus* and *L. graecus*, and with 20A06 in *L. albus* (Supplementary Figures S4–S6). 

The Lang13 chromosome was marked by three clones (11G20, 8C03, 51F15) and was visualized in other lupins, either by the clone 8C03 in *L. micranthus* (Lmic13) or 51F15 in seven other species: *L. hispanicus*, *L. albus*, *L. graecus*, *L. atlanticus*, *L. digitatus*, *L. cosentinii*, and *L. pilosus*. However, in *L. luteus*, we identified two chromosomes (Llut13, Llut13′) that carried 51F15 (Supplementary Figure S3). Chromosome markers of Lang19 have been used to show that BACs 83F23 and 67C07 were mapped in one Lang19-like chromosome in seven species, as *L. cryptanthus*, *L. hispanicus*, *L. micranthus*, *L. digitatus*, *L. cosentinii*, *L. pilosus*, and *L. palaestinus*, and one BAC 83F23 was found in *L. luteus*. Surprisingly, all of the clones in *L. atlanticus* were mapped with patterns that were typical for *L. angustifolius*. Also, in the case of the Lang20-specific clones, we observed that both 17B07 and 83F23 were localized in one Lang20-like chromosome of species such as *L. albus*, *L. micranthus*, *L. atlanticus*, *L. digitatus*, *L. cosentinii*, and *L. pilosus*, but only BAC 17B07 hybridized with Lhis20 and Lgra20 in *L. hispanicus* and *L. graecus*, respectively. All of the three Lang20-specific clones (17B07, 83F23, 1M23) were represented in *L. cryptanthus* and *L. luteus* (Supplementary Figures S2 and S3). In the Lang04-like chromosome, four lupins had a chromosome that carried both clones, but the other four only had one of them, BAC 47P22. Furthermore, within Lang10-like chromosomes, clone 77C13 was mapped in one chromosome of six lupin species, while its colocalization with clone 57K22 was detected only in two species: *L. cryptanthus* and *L. micranthus* (Supplementary Figures S2 and S7).

## 4. Discussion

### 4.1. Lupin Chromosomes Reveal Their Puzzling Evolution

Our analysis shows that multiple chromosomal rearrangements were responsible for the chromosome variations in OWLs. We propose a model that involves complex changes that shaped the variations in chromosome number and/or structure. We assume that the mechanisms driving these differences involved DNA gain or loss through insertion/duplication or deletion. In addition, chromosome changes can also be derived from rearrangements within or between chromosomes, through insertion or translocation [27]. In particular, we hypothesized that at least four types (I–IV) of rearrangements have impacted on lupin chromosome changes. Possible changes of Lang06-like and Lang17-like chromosomes (Figure 6) might be explained by translocations of both arms of Lang chromosomes, to lead to two Lang06-like chromosomes in *L. luteus*, *L. albus*, *L. graecus* and *L. micranthus*, and two Lang17-like chromosomes in *L. pilosus*. However, these chromosomes might also have arisen from chromosome breakage, and have then undergone chromosome fusion-fission. We have called these possible rearrangements type I. Chromosomal changes within type II might encompass translocation of arm A and arm B to three chromosomes, which would result in two chromosomes carrying arm A, and one with arm B. However, translocation of these arms might have involved two chromosomes (one with arm A, the other with arm B), with the subsequent duplication of chromosome-carrying clones from arm A. Additionally, chromosome breakage might occur as well, together with the translocation of arm A or the duplication of the entire chromosome-carrying clones from arm A. This type of change was exclusively seen for the Lang06-like chromosome in *L. pilosus* and the Lang17-like chromosomes in *L. digitatus*, where three chromosomes (Lpil06, Lpil06′, Lpil06′′ Ldig17, Ldig17′, Ldig′′; respectively) were identified. Type III rearrangements were seen for the Lang06-like chromosome in *L. hispanicus*, *L. digitatus* and *L. cosentinii*, and for the Lang17-like chromosome in *L. palaestinus*. They show changes that underlie either translocation of one of the chromosome arms (arm A) or chromosome breakage, with the loss of one of the chromosome arm (arm B). The fourth type of rearrangement (type IV) was detected uniquely in the Lang06-like chromosomes of *L. palaestinus*. It might have evolved via multiple changes, such as translocation of arm A to two chromosomes, translocation of arm A to one chromosome and then duplication of the entire chromosome, or chromosome breakage and loss of one chromosome carrying clones from B and then duplication of the entire chromosome with translocation of arm A. In *L. cryptanthus* (2n = 40) and *L. atlanticus* (2n = 38), Lang06-like chromosomes were identified with patterns typical to *L. angustifolius*, beyond ‘repetitive’ clone 51D03 in *L. atlanticus*. Changes within the Lang17-like chromosome were noted only in three species: *L. digitatus*, *L. pilosus* and *L. palaestinus*. In other lupins, the BAC-FISH patterns were visualised as for *L. angustifolius*, with the exception of the ‘repetitive’ BAC 111G03 in *L. cryptanthus*.

Chromosome translocation changes between lupins can be explained in a similar way to those identified between *Phaseolus vulgaris* and *Vigna unguiculata*, where two BACs from two chromosomes of *P. vulgaris* were mapped to one chromosome of *V*. *unguiculata* [28]. However, the possibility of chromosome breakage and then chromosome fusion-fission in lupins, which could well occur in centromere regions and lead to chromosome reduction, needs to be further demonstrated using additional centromeric markers. Despite the tendency of chromosome reduction as a general evolutionary event, the mechanisms of these events are unclear in lupins [17]. Dysploidy via chromosome fusion-fission has been widely recognized in grasses, such as the *Brachypodium* genus [29,30], as well as in Brassicaceae [31], but in legumes it has only been seen in *Phaseolus leptostachyus*, with its chromosome number 2n = 20 [32]. On the other hand, ascending or descending aneuploidy via duplication or losses of individual chromosomes might also have a pivotal role in plants [1]. It has been shown that descending aneuploidy occurred mostly in early diverging papilionoid lineages (to which lupins belong), especially in species with low basic chromosome numbers [33]. Aneuploidy is exemplified in the *Arachis* genus (2n = 2x = 18) [34], *Lathyrus* [35], and *Medicago* sections [36]. Both dysploidy and aneuploidy in lupins cannot be ruled out, considering that polyploidy is associated with chromosome number reduction in core papilionoids [33,37]. However, aneuploidy complex series [38] and duplication and triplication [39] have been already suggested as crucial players in lupin genome variations. Importantly, Drummond [38] noted that an additional evolutionary mechanism like allopolyploidy might be unique to OWLs. They also assumed that the diversification within NWLs with stable chromosome numbers of 2n = 36 or 2n = 48 resulted from ecological speciation chromosome changes related to ploidy, with the exception of eastern North American lupins, with chromosome 2n = 52.

**Figure 7:**
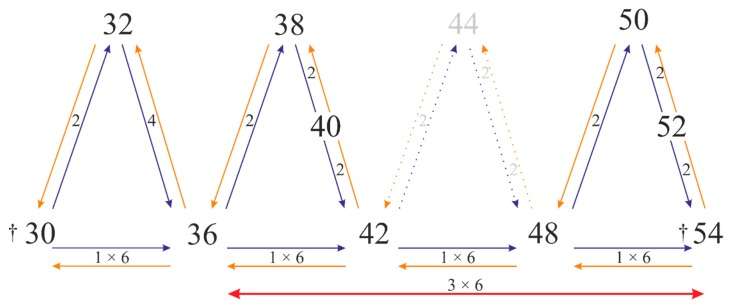
Hypothetical pattern of direction of lupin chromosome evolution through polyploidy and aneuploidy events. † describes extinct species; considering x = 6 the 1 × 6 corresponds to one round of whole genome duplication (WGD), while 3 × 6 three rounds of WGD; number 2 (4) indicates the aneuploidy events.

Thus, we address the question whether the course of lupin karyotypes followed from karyotype 2n = 52 to 2n = 36. In theory, species with 2n = 52 can evolve from ‘ancestral’ species with 2n = 54. There is no data about lupin with 2n = 54 chromosomes; however, they might be extinct. Furthermore, there have not been species with 2n = 44, 46 and 48 described within OWLs, while in NWLs, species with 2n = 48 are widely seen [40,41]. It can be suggested that species with 2n = 54 were the ‘ancestral’ ones, whereby the species with lower chromosome numbers might have undergone three rounds of whole genome duplication (WGD), to result in 2n = 48, 2n = 42 and 2n = 36, with the assistance of aneuploidy giving chromosome numbers 2n = 52, 50, 40, 38, 32. In this sense, lupin karyotypes might also have arisen due to aneuploidy, as their chromosome number is often different from the simple multiple of the basic chromosome number, like in *L. cosentinii* (2n = 32), *L. atlanticus* (2n = 38), *L. angustifolius* (2n = 40), *L. albus* (2n = 50), and *L. luteus* (2n = 52). We now present here our hypothetic model of lupin chromosome evolution, which is based on the polyploidy events supplemented by the likely aneuploidy events (Figure 7). We assume that the basic chromosome number in OWLs is x = 6. The idea of the basic chromosome number x = 6 is consistent the same base number considered for NWLs [41] and the ancestral rosid karyotype model [42], but contrary to comparative phylogenetic analyses, where the ancestral basic chromosome number for lupins as a member of genistoids was postulated to be x = 9 [43].

Of importance, multiple and various rearrangements in chromosome structures support our hypothesis regarding variations in the lupin chromosomes. Lupin chromosome variation can be explained by different genome organization and evolutionary relationships between the reference *L. angustifolius* and the related lupins. Of note, the identification of chromosome changes was not directly linked to the number of clones used for comparative mapping. Four clones were enough to detect structural changes for Lang03 and Lang11 in all of the lupins. However, the localization of four clones in the Lang08-like chromosomes illustrated structural changes in the species analyzed, and chromosome number rearrangements only in *L. micranthus*. Furthermore, it can also be considered that an individual chromosome might have undergone its specific and independent evolutionary changes. 

We realize that a cytogenetically based perspective can underestimate the evolutionary events that have shaped plant genomes. Lupins belong to the genistoid clade, which is known as one of the most complex groups in the facet of polyploidy, and one of the weakest supported groups in core papilionoids [33]. The advent of lupin genome sequencing data and the availability of extensive genomic resources should be accomplished in the future. Additionally, the power of informative cytogenetic mapping to track genome duplication or extensive synteny among species can be increased while suitable model species are adopted, like model soybean and common bean [44], or *Arabidopsis thaliana* and other species within Brassicaceae [45,46]. 

Thus, for further comparative chromosome analyses, we designated *L. digitatus*, *L. pilosus*, and *L. palaestinus* as the reference species to track chromosome evolution within lupins. These are species showing chromosome changes of both Lang06- and Lang17-like chromosomes (Figure 6). Interestingly, these species have chromosome numbers that directly correspond to the basic chromosome number x = 6. Phylogeny analyses indicated that *L. digitatus* and *L. palaestinus* are the youngest ones, with diversification times estimated at about 0.5 mya. The diversification time of *L. pilosus* was estimated as about 3 mya [47]. Although *L. pilosus* and *L. palaestinus* share the same chromosome number, they differ in the divergence times and different types of chromosome changes among the Lang06-like and Lang17-like chromosomes. This sheds light on different evolutionary patterns of these two species with 2n = 42, and also *L. digitatus* with 2n = 36. It can be hypothesized that *L. digitatus* and *L. palaestinus* have undergone at least one common round of WGD, while *L. pilosus* might have been driven by different WGD.

On the other hand, there is a lupin group with chromosome number 2n = 52 or 2n = 50. In contrast to *L. digitatus*, *L. micranthus* (2n = 52), and *L. albus* (2n = 50) are two of the oldest species within the OWLs, with divergence about 7 mya and 7.5 mya, respectively [47]. It is intriguing the way species carrying chromosome 2n = 50-52 might have evolved. We wonder whether species with chromosome number 2n = 52, like *L. micranthus*, *L. luteus*, and *L. hispanicus*, might have evolved through different evolutionary events, as *L. luteus* and *L. hispanicus* diverged about 2.0 mya [47,48]. Consider also that *L. micranthus* and *L. luteus* have the smallest (0.98 pg/2C DNA) and largest (2.44 pg/2C DNA) genomes, respectively, in OWLs. On the other hand, *L. hispanicus* showed Lang06-like chromosome changes that were the same as *L. digitatus*. To highlight the complexity of this lupin genome variation, we note that *L. digitatus* and *L. hispanicus* share the highest number of clones that were mapped as ‘repetitive’. They have different chromosome numbers and diversification rates, and they are geographically separated. *L. digitatus* is distributed mainly in the northern part of Africa (Sahara and Egypt), and *L. hispanicus* is in north-western Spain, Portugal, Greece, and Turkey [14]. 

### 4.2. Cytogenetic Resources for Lupin Genome Analyses

Here we have demonstrated that the integration of whole genome sequencing and chromosome mapping through BAC-FISH can be successfully used to determine the genome assembly of the genome of *L. angustifolius*. This integrative approach has allowed us to construct a comprehensive chromosomal map that might be erroneously interpreted, if only the sequence assembly was to be applied, as it was applied to precisely assign the chromosomes and pseudomolecules of *Brachypodium distachyon* [49] and cotton [50]. As a consequence, we can finally integrate the unassigned linkage groups, as was shown for cluster-2, which has QTLs for flowering time [51] and flowering time gene homologue markers [39]. This ideogram developed for *L. angustifolius* defines the complete genomic map through the high number of chromosome markers that were assigned using whole BAC sequences.

For example, in our previous study [17], Lang08 carrying clones BACs 84D22, 111B88, 142C04, and 142D13 were mapped to scaffold AOCW01048841of the *L. angutifolius* genome published by Yang, et al. [52], while in the present study it was localized in the pseudomolecule NLL17 of the *L. angutifolius* genome [20]. Even so, implementation of FISH with these BACs as probes, excludes them as markers of Lang17 [23]. In this case, we established them as markers for Lang08 by the supportive ideogram provided by Wyrwa et al. [24]. Conversely, some BACs were localized in pseudomolecules, while they were not mapped in linkage groups. Similarly, we were able to saturate the pseudomolecules NLL13 by clones 8C03, which remained unmapped in the genetic map [23]. In this way, the chromosome order was also reshuffled, as was done for Lang19 and Lang07.

As the number of sequenced genomes is rising rapidly, there is the need to produce the appropriate tools to establish high quality reference genomes among plants studied that can be used for analyses of genomic variation. Furthermore, and especially in the case of some complex genomes, BAC sequencing can still contribute to improved genome sequencing carried out by NGS methods [53], and can remain as an additional ‘check point’, even if advanced techniques like BioNano optical mapping and Hi-C sequencing are implemented for the comparative assembly of genomes [54]. 

In *Lupinus* species, only the whole genome sequence of *L. angustifolius* is available so far. Thus, the sequence-comparison-based evolutionary research in this group of plants is impossible, to date. In addition, the other genetic resources, such as genetic maps and reliable chromosome-specific markers, are also rather limited. Hence, the unambiguous BAC markers developed can help guide comparative research in lupins and can also be useful to distinguish crop and wild genotypes (e.g., *L. albus* from *L. graecus*). In addition, we have provided markers for individual chromosomes of *L. cosentinii* (2n = 32). Further reciprocal BAC-FISH within this species should provide the completed ideogram of its karyotype. Additionally, in *L. albus* (2n = 50), these markers can be used as anchors to integrate the pseudomolecules and chromosomes of the already sequenced white lupin genome (Peret laboratory; personal communication). 

A few BAC clones per chromosome, similar to the number we used for particular lupin chromosomes, were successful used to compare karyotypes of *Phaseolus* [10] and to discover dysploidy in this genus, as mentioned above [32]. Considering the high chromosome numbers in lupins, there is the suggestion to track particular chromosome of complement in more detailed pathways, either by providing higher numbers of BAC probes for FISH, as were generated for chromosome 6 in tomato [12] and chromosome 9 in maize [55]. The most advanced comparative studies require the use of high numbers of clones assigned to particular chromosomes, to track conserved chromosomal syntenies [56], reveal hypothesized ancestral chromosome numbers, and reconstruct the course of chromosome rearrangements during evolution [57,58]. An alternative approach is to implement the promising oligo-painting method [59] to investigate changes at different chromosome or chromosome-arm scales. 

## 5. Conclusions

We have shown that some Lang-like chromosomes retain the same BAC-FISH patterns, while others expand with multiple rearrangements. We cannot determine the direction of chromosomal changes that shaped these lupin genomes, as we have assumed that any individual chromosome might undergone independent evolution. However, a tendency of the chromosome number to be reduced through translocation and/or breakage in the lupin genus was seen (mainly in the Lang06-like and Lang17-like chromosomes, and a few other chromosomes). We propose *L. digitatus* (2n = 36), *L. pilosus* (2n = 42) and *L. palaestinus* (2n = 42) as reference species for comparative evolutionary analyses here. Given possibly three independent WGD in lupins, together with the predominant chromosomal data of chromosome numbers and phylogenetic analyses, we hypothesize that the lupin basic chromosomes with x = 6 arose from a reduction from 2n = 54, with assistance of aneuploidy. *Lupinus* are considered as polyploids, like for many agricultural crops. However, the evolutionary relationships between lupins in terms of their complex polyploidy origin are neither evident nor simple.

## Figures and Tables

**Table 1:** General characteristics of the *Lupinus* species used in this study.

Group	Section	Species	Accession	Chromosome Number (2n)	Genome Size (pg/2C DNA)
Smooth-seeded	Angustifolius	*L. angustifolius*	cv. ‘Sonet’ *	40	1.89
		*L. cryptanthus*	96361 *	40	1.86
	Luteus	*L. luteus*	cv. ‘Talar’ *	52	2.44
		*L. hispanicus*	96385 *	52	2.15
	Albus	*L. albus*	cv. ‘Boros’ *	50	1.16
		*L. graecus*	95601 *	50	1.13
	Micranthus	*L. micranthus*	98552 *	52	0.98
Rough-seeded	Atlanticus	*L. atlanticus*	98401 *	38	1.61
		*L. digitatus*	PI 660697 **	36	1.37
		*L. cosentinii*	98452 *	32	1.42
	Pilosus	*L. pilosus*	98653 *	42	1.36
		*L. palaestinus*	98605 *	42	1.39

* Polish *Lupinus* Gene Bank, Breeding Station Wiatrowo, Poznan Plant Breeders Ltd., Poland ** US Department of Agriculture, USA.

**Table 2:** Bacterial artificial chromosome (BAC) clones used for the chromosome and pseudomolecule integration in *L. angustifolius*.

Assignment	Chromosome (Lang)	Pseudomolecule (NLL)	Pseudomolecule (bp)	BAC ID	BAC Data	GenBank Accession
*Lang01*	01	01	1..36 457 581	43C18	**WBS**	MK650088
		13				
Lang02	02	02	1..24 697 652	120E23	**WBS**	MK650090
Lang03	03	03	1..30 153 019	84A06	**WBS**	MK650076
	03	03		57J20	WBS	HE804810.1
	03	03		28O01	3′ STS	GF112056.1
	03	03		137N08	**WBS**	MK650084
Lang04	04	04	1..27 333 975	47P22	**WBS**	MK650072
	04	04		131K22	5′ BES	KU678257.1
	04	04			3′ BES	KU678256.1
Lang05	05	05	1..26376911	131K15	**WBS**	MK650081
	05	05		4G15	WBS	HE804808.1
Lang06	06	06	1..33 111 450	44J16	WBS	KX298066
	06	06		76K16	**WBS**	MK650073
	06	06		80B11	WBS	HE804812.1
	06	06		51D03	**WBS**	MK650071
	06	06		127N17	WBS	KU678223.1
*Lang07*	Not assigned	07	1..19 782 170	2B03	WBS	KX298069
*Lang08*	08	17	1...25 521 646	84D22	WBS	KX298065
	08	17		111B08	WBS	KX298071
	08	17		142C04	WBS	KX298073
	08	17		142D13	WBS	KX298074
Lang09	09	09	1..21 753 712	15P08	5′ BES	AB809174.1
		09			3′ BES	AB809173.1
	09	09		59J08	**WBS**	MK650082
Lang10	10	10	1...16 341 955	77C13	**WBS**	MK650077
	10	10		57K22	**WBS**	MK650069
Lang11	11	11	1..35 963 958	97D16	**WBS**	MK650085
	11	11		36L23	**WBS**	MK650075
	11	11		20A06	**WBS**	MK650080
	11	11		60F02	WBS	MK045265
Lang12	12	12	1..19 065 701	94P05	**WBS**	MK650086
*Lang13*	Not analyzed	Many	1..17 820 680	11G20	**WBS**	MK650079
	Not assigned	13		8C03	WBS	KX298063
	Not analyzed	13		51F15	WBS	MK045272
*Lang14*	14	03	1...16 251 777	138N02	**WBS**	MK650089
	14	14		115C21	5′ BES	HR864196.1
		12			3′ BES	AB809318.1
	Not analyzed	14		9K06	WBS	MK045273
Lang15	15	15	1...20 964 703	5L11	3′ STS	GF112057.1
	15			134F01	**WBS**	MK650070
Lang16	16	Many	1..20 786 881	115G22	5′ BES	AB811328.1
		Many			3′ BES	AB811323.1
	16	Many		112E01	5′ BES	AB809308.1
		Many			3′ BES	AB809307.1
	16	Many		72O21	5′ BES	AB809272.1
	16	20		8A03	5′ BES	AB809167.1
		Many			3′ BES	AB809166.1
	16	16		87N22	**WBS**	MK650087
Lang17	17	17	1..21 299 880	111G03	WBS	KX298064
	17	17		136C16	WBS	KX298072
	17	17		3B18	WBS	KX298070
Lang18	18	18	1..16 588 007	68H10	WBS	KU678221
*Lang19*	19	19	1..18 159 812	67C07	**WBS**	MK650078
	19	19		83F23	5′ BES	KU678304.1
		19			3′ BES	KU678303.1
	07	19		74I10	**WBS**	MK650074
*Lang20*	20	20	1..21 988 590	17B07	WBS	HF937076.1
	20	20		83C06	**WBS**	MK650083
	Cluster-2	20		1M23	WBS	KX298067

BAC: bacterial artificial chromosome; BES: BAC-end sequences; WBS: whole BAC sequences; in bold when provided in this study. Lang, NLL, results of chromosome, and pseudomolecule integration, respectively, with reassigned chromosome numbers in italics.

**Table 3:** Characteristics and localizations of the BAC clones used for the comparative BAC-FISH.

No.	BAC ID	Lcry	Llut	Lhis	Lalb	Lgra	Lmic	Latl	Ldig	Lcos	Lpil	Lpal
	**Lang01**	**1-R**	**1-R**	**1-R**	**1-R**	**1-R**	**1-R**	**1-R**	**1-R**	**1-R**	**1-R**	**1-R**
1	43C18	R	R	R	R	R	R	R	R	R	R	R
	**Lang02**	**S**	**S**	**S**	**2-R**	**2-R**	**S**	**S**	**2-R**	**S**	**S**	**S**
2	120E23	S	S	S	R	R	S	S	R	S	S	S
	**Lang03**	**3=4=6; 5-R**	**3-4-5-6-R**	**3-4-5-6-R**	**3=4=6; 5-R**	**3=4; 5-6-R**	**3=6; 4-5-R**	**3-4-5-6-R**	**3-5-6-R**	**3=6; 4-5-R**	**3=6; 4-5-R**	**3=4=6; 5-R**
3	84A06	S	R	R	S	S	S	R	R	S	S	S
4	57J20	S	R	R	S	S	R	R	N/D	R	R	S
5	28O01	R	R	R	R	R	R	R	R	R	R	R
6	137N08	S	R	R	S	R	S	R	R	S	S	S
	**Lang04**	**7=8**	**7-8-R**	**7=8**	**7; 8-R**	**7=8**	**7; 8-R**	**7-8-R**	**7-8-R**	**7; 8-R**	**7; 8-R**	**7=8**
7	47P22	S	R	S	S	S	S	R	R	S	S	S
8	131K22	S	R	S	R	S	R	R	R	R	R	S
	**Lang05**	**9=10**	**9=10**	**9-10-R**	**9=10**	**9=10**	**9=10**	**9=10**	**9=10**	**9=10**	**9=10**	**9=10**
9	131K15	S	S	R	S	S	S	S	S	S	S	S
10	4G15	S	S	R	S	S	S	S	S	S	S	S
	**Lang06**	**11=12=13=15=16**	**11=13 #14; 12-15-R**	**13; 11-12-14-15-R**	**12=13 # 14; 11-15-R**	**12=13 # 14; 11-15-R**	**12=13 # 14=15**	**11=12=13=15; 14-R**	**12=13; 11-14-15-R**	**13; 12-14-15-R**	**12 # 13 # 14=15**	**12=13#12′; 11-14-15-R**
11	44J16	S	S	R	R	R	N/D	S	R	N/D	N/D	R
12	76K16	S	R	R	S	S	S	S	S	R	S	S, S′
13	80B11	S	S	S	S	S	S	S	S	S	S	S
14	51D03	S	S	R	S	S	S	R	R	R	S	R
15	127N17	S	R	R	R	N/D	S	S	R	R	S	R
	**Lang07**	**S**	**S**	**S**	**S**	**S**	**16-R**	**S**	**16-R**	**S**	**S**	**S**
16	2B03	S	S	S	S	S	R	S	R	S	S	S
	**Lang08**	**17=18=19=20**	**18=20; 17-19-R**	**17-18-19-20-R**	**17-18-19-20-R**	**17-18-19-20-R**	**17=19 # 18; 20-R**	**17-18-19-20-R**	**17-18-19-20-R**	**17=18; 19-20-R**	**17=18; 19-20-R**	**17-18-19-20-R**
17	84D22	S	R	R	R	R	S	R	R	S	S	R
18	111B08	S	S	R	R	R	S	R	R	S	S	R
19	142C04	S	R	R	R	R	S	R	R	R	R	R
20	142D13	S	S	R	R	R	R	R	R	R	R	R
	**Lang09**	**22; 21-R**	**22; 21-R**	**22; 21-R**	**22; 21-R**	**22; 21-R**	**22; 21-R**	**22; 21-R**	**22; 21-R**	**22; 21-R**	**22; 21-R**	**22; 21-R**
21	15P08	R	R	R	R	R	R	R	R	R	R	R
22	59J08	S	S	S	S	S	S	S	S	S	S	S
	**Lang10**	**23=24**	**23; 24-R**	**23-24-R**	**23; 24-R**	**23-24-R**	**23=24**	**23; 24-R**	**23; 24-R**	**23-24-R**	**23-24-R**	**23-24-R**
23	77C13	S	S	R	S	R	S	S	S	R	R	R
24	57K22	S	R	R	R	R	S	R	R	R	R	R
	**Lang11**	**25=26=27=28**	**26; 25-27-28-R**	**25=26; 27-28-R**	**25=27; 26-28-R**	**25=26; 27-28-R**	**26; 25-27-28-R**	**25; 26-27-28-R**	**26-27-28-R**	**26; 25-27-28-R**	**26; 25-27-28-R**	**25; 27-28-R**
25	97D16	S	R	S	S	S	R	S	N/D	R	R	S
26	36L23	S	S	S	R	S	S	R	R	S	S	N/D
27	20A06	S	R	R	S	R	R	R	R	R	R	R
28	60F02	S	R	R	R	R	R	R	R	R	R	R
	**Lang12**	**29-R**	**S**	**29-R**	**S**	**S**	**29-R**	**29-R**	**29-R**	**29-R**	**29-R**	**S**
29	94P05	R	S	R	S	S	R	R	R	R	R	S
	**Lang13**	**31=32; 30-R**	**32 # 32′; 30-31-R**	**32; 30-31-R**	**32; 30-31-R**	**32; 30-31-R**	**31; 30-32-R**	**32; 30-31-R**	**32; 30-31-R**	**32; 30-31-R**	**32; 30-31-R**	**30-31-32-R**
30	11G20	R	R	R	R	R	R	R	R	R	R	R
31	8C03	S	R	R	R	R	S	R	N/D	R	R	R
32	51F15	S	S, S′	S	S	S	R	S	S	S	S	R
	**Lang14**	**33=35; 34-R**	**33-34-35-R**	**33-34-35-R**	**33-34-35-R**	**35; 33-34-R**	**35; 33-34-R**	**35; 33-34-R**	**33-34-35-R**	**35; 33-34-R**	**35; 33-34-R**	**33-34-35-R**
33	138N02	S	R	R	R	R	R	R	R	R	R	R
34	115C21	R	R	R	R	R	R	R	R	R	R	R
35	9K06	S	R	R	R	S	S	S	R	S	S	R
	**Lang15**	**37; 36-R**	**36-37-R**	**36-37-R**	**36-37-R**	**36-37-R**	**37; 36-R**	**36-37-R**	**37; 36-R**	**37; 36-R**	**37; 36-R**	**36-37-R**
36	5L11	R	R	R	R	R	R	R	R	R	R	R
37	134F01	S	R	R	R	R	S	R	S	S	S	R
	**Lang16**	**38=39=40=41; 42-R**	**38=39=40=41; 42-R**	**38=39=40=41; 42-R**	**38=39=40=41; 42-R**	**38=39=40=41; 42-R**	**38=39=40=41; 42-R**	**38=39=40=41; 42-R**	**38=39=40=41; 42-R**	**38=39=40=41; 42-R**	**38=39=40=41; 42-R**	**38=39=40=41; 42-R**
38	115G22	S	S	S	S	S	S	S	S	S	S	S
39	112E01	S	S	S	S	S	S	S	S	S	S	S
40	72O21	S	S	S	S	S	S	S	S	S	S	S
41	8A03	S	S	S	S	S	S	S	S	S	S	S
42	87N22	R	R	R	R	R	R	R	R	R	R	R
	**Lang17**	**43=45; 44-R**	**43=44=45**	**43=44=45**	**43=44=45**	**43=44=45**	**43=44=45**	**43=44=45**	**43 # 44=45 # 45′**	**43=44=45**	**43 # 44=45**	**44=45; 43-R**
43	3B18	S	S	S	S	S	S	S	S	S	S	R
44	111G03	R	S	S	S	S	S	S	S	S	S	S
45	136C16	S	S	S	S	S	S	S	S, S′	S	S	S
	**Lang18**	**46-R**	**46-R**	**46-R**	**46-R**	**46-R**	**46-R**	**46-R**	**46-R**	**46-R**	**46-R**	**46-R**
46	68H10	R	R	R	R	R	R	R	R	R	R	R
	**Lang19**	**47=48; 49-R**	**47; 48-49-R**	**47=48; 49-R**	**47-49-R**	**47-48-49-R**	**47=48; 49-R**	**47=48=49**	**47=48; 49-R**	**47=48; 49-R**	**47=48; 49-R**	**47=48; 49-R**
47	67C07	S	S	S	R	R	S	S	S	S	S	S
48	83F23	S	R	S	N/D	R	S	S	S	S	S	S
49	74I10	R	R	R	R	R	R	S	R	R	R	R
	**Lang20**	**50=51=52**	**50=51=52**	**50; 51-52-R**	**50=51; 52-R**	**50; 51-52-R**	**50=51; 52-R**	**50=51; 52-R**	**50=51; 52-R**	**50=51; 52-R**	**50=51; 52-R**	**50=51; 52-R**
50	17B07	S	S	S	S	S	S	S	S	S	S	S
51	83C06	S	S	R	S	R	S	S	S	S	S	S
52	1M23	S	S	R	R	R	R	R	R	R	R	R

BAC clones are individually numbered from 1 to 52 and grouped with correspondence to *L. angustifolius* chromosomes (Lang). There are four possible BAC-FISH mapping results: S, ‘single’ BAC; S, S′, BAC mapped into two loci of two chromosomes; R, ‘repetitive’ BAC; N/D, not detected. For example: (a) BAC clone no. 2—120E23 corresponding to Lang02 is ‘single’ (S) in Lcry, but ‘repetitive’ (R) in Lalb; (b) 3=4; 5-6-R means that BACs 84A06 and 57J20 were mapped as ‘single’ in the same chromosome, but both clones 28O01 and 137N08 were ‘repetitive’; (c) 11=13 #14; 12-15-R means that BACs 11 and 13 were mapped in the same chromosome, while BAC 14 was mapped in different chromosome, both BACs 12 and 15 were ‘repetitive’.

## Supplementary Materials

The following are available online at https://www.mdpi.com/2073-4425/10/4/259/s1, Table S1: Characterization of the BAC clone position in the pseudomolecules of the *L. angustifolius* genome (Hane et al. 2017), Figure S1: Origin and distribution of Old World lupins. The geographical range is based on Gladstones (1998), with minor editorial modifications, Figure S2–S12: Ideograms of individual chromosomes in lupins. (S2) *L. cryptanthus*. (S3) *L. luteus*. (S4) *L. hispanicus* (S5) *L. albus*. (S6) *L. graecus*. (S7) *L. micranthus*. (S8) *L. atlanticus*. (S9) *L. digitatus*. (S10) *L. cosentinii*. (S11) *L. pilosus*. (S12) *L. palaestinus*. BAC markers are indicated by rectangles with unique colors, Figure S13: Schematic visualization of the results of comparative BAC-FISH among lupins in this study. BAC clones mapped either as ‘single’ or ‘repetitive’ were indicated by single rectangle or triple rectangle with its unique color, respectively. ‘Repetitive’ clones were described by the R at the end of their names, for example the clone named as 43C18_R is ‘repetitive’, while the clone 43C18 is a ‘single’ one. The colors of rectangles were used according to the RGB code. All chromosomes were drawn to scale to *L. angustifolius* chromosomes and BAC positions in *L. angustifolius* genome (Hane et al. 2017), Figure S14: Diagrams with examples of the changes in chromosome structure, deduced according to the presence/absence of ‘single’ BACs in particular Lang-like chromosomes.
